# Assessment of the Anti-Amyloidogenic Properties of Essential Oils and Their Constituents in Cells Using a Whole-Cell Recombinant Biosensor

**DOI:** 10.3390/brainsci14010035

**Published:** 2023-12-29

**Authors:** Electra Stylianopoulou, Anastasia Daviti, Venetia Giourou, Eleni Gerasimidi, Anastasios Nikolaou, Yiannis Kourkoutas, Maria E. Grigoriou, Katerina E. Paleologou, George Skavdis

**Affiliations:** 1 Laboratory of Developmental Biology & Molecular Neurobiology, Department of Molecular Biology & Genetics, Democritus University of Thrace, 68100 Alexandroupolis, Greece; ilstylian@mbg.duth.gr (E.S.); daviti.anastasia@gmail.com (A.D.); elgerasimidi@gmail.com (E.G.); mgrigor@mbg.duth.gr (M.E.G.); 2 Laboratory of Molecular Regulation & Diagnostic Technology, Department of Molecular Biology & Genetics, Democritus University of Thrace, 68100 Alexandroupolis, Greece; venetiag69@gmail.com; 3 Laboratory of Applied Microbiology & Biotechnology, Department of Molecular Biology & Genetics, Democritus University of Thrace, 68100 Alexandroupolis, Greece; anikol@mbg.duth.gr (A.N.); ikourkou@mbg.duth.gr (Y.K.)

**Keywords:** α-synuclein, Parkinson’s disease, split-luciferase assay, *Gaussia princeps*, essential oils, citron, sage, linalool, eucalyptol

## Abstract

Essential oils exhibit numerous medicinal properties, including antimicrobial, anti-inflammatory and antioxidant effects. Recent studies also indicate that certain essential oils demonstrate anti-amyloidogenic activity against β-amyloid, the protein implicated in Alzheimer’s disease. To investigate whether the anti-aggregating properties of essential oils extend to α-synuclein, the protein involved in Parkinson’s disease, we constructed and employed a whole-cell biosensor based on the split-luciferase complementation assay. We validated our biosensor by using baicalein, a known inhibitor of α-synuclein aggregation, and subsequently we tested eight essential oils commonly used in food and the hygienic industry. Two of them, citron and sage, along with their primary components, pure linalool (the main constituent in citron essential oil) and pure eucalyptol (1,8-cineole, the main constituent in sage essential oil), were able to reduce α-syn aggregation. These findings suggest that both essential oils and their main constituents could be regarded as potential components in functional foods or incorporated into complementary Parkinson’s disease therapies.

## 1. Introduction

Essential oils (EOs) are aromatic plant-derived liquid extracts that can be obtained from all parts of plants, including their flowers, fruits and seeds [1]. They can be extracted by various methods, such as distillation (hydrodistillation, steam distillation, etc.), solvent extraction and solvent-free microwave extraction] [1], depending on the purpose of their final use. It should be noted that the extraction method and protocol can affect their chemical profile and quality [1,2,3]. EOs are a complex blend of chemical constituents, ranging in number from 20 to 60 [2], that can be grouped into three classes: terpenes (monoterpenes), terpenoids (isoprenoids) and aromatic compounds (aldehyde, alcohol, phenol and more) [1,2].

EOs have been reported to exhibit antibacterial, antifungal, antiviral [4,5], anti-inflammatory, antioxidant, insect repelling [5] and insecticide [6] properties. Due to all these activities, EOs are utilized in pharmaceutical, food and hygienic industries and in agriculture, medicine and dentistry [2], while recent studies advocate neuroprotective and anti-amyloidogenic activity for certain EOs and their components. More specifically, the EO of *Artemisia absinthium* (wormwood) was shown to inhibit β-amyloid (Aβ) aggregation in vitro [7], the EO of *Cinnamomum Zeylanicum* (cinnamon) inhibited the self-induced and Cu^2+^-induced aggregation of Aβ_1–42_ in vitro [8], while the EO of *Rosa Setate x Rosa Rugosa* (Kushui rose) decreased Aβ oligomers and deposition in a *C. elegans* Alzheimer’s disease (AD) model [9]. EOs components including linalool, (*E*)-cinnamaldehyde and (*E*)-cinnamyl acetate have been also shown to suppress Aβ aggregation [10,11]. Given that acetylcholinesterase has a pro-aggregating effect on Aβ (reviewed in [12]), it is noteworthy that several EOs or their components have anticholinesterase activity [8,10,13,14,15,16,17,18]. Additionally, there are EOs that can also inhibit tau phosphorylation [19,20]. Moreover, several lines of evidence support that EOs and their components can attenuate Aβ-induced toxicity and impairments. The EO of *Pinus halepensis* (Aleppo pine) has been demonstrated to alleviate Aβ_1–42_-induced memory dysfunction and toxicity in rat brains [15], whereas *Chamaecyparis obtuse* (Japanese cypress) EO decreased Aβ-related neuronal apoptosis and improved the behavioral deficits seen in Aβ_1–40_-injected rats [21]. *Lavandula angustifolia* (lavender) and *Coriandrum sativum* (coriander) EOs are also neuroprotective, as treatment of cells exposed to oligomeric Aβ_1–42_ with each of these EOs resulted in an increase in cell viability, a decrease in oxidative stress and a reduction in the expression of the pro-apoptotic casapce-3 [22]. It has been also demonstrated that EOs can improve AD-related deficits through other routes, including increased expression of neurotrophic factors [23] and modulation of autophagy [24]. There is also ample evidence that EOs components, such as linalool, (−)-carveol, possess antioxidant [11,25,26], anti-inflammatory [11,25] and anti-anxiety properties [11].

Similarly to Aβ that is considered to play a key role in AD pathogenesis (reviewed in [27]), α-synuclein (α-syn) aggregation is believed to be central to the pathogenesis of a series of neurodegenerative diseases collectively referred to as “synucleinopathies” (reviewed in [28]), with Parkinson’s disease (PD) being the most prominent. Given that PD and related disorders are incurable at present, the utilization of EOs could potentially be used as a complementary therapy for improving patients’ quality of life [29], despite the fact that their effectiveness against certain aspects of neurodegenerative diseases (dementia, anxiety and agitation) still remains unclear [30,31,32,33,34]. There is increasing evidence that EOs and their constituents can have an impact against PD due to neuroprotective [35,36,37], antioxidant [35,36,37], anti-inflammatory [36], anti-apoptotic [35,36] and proteasome- [35] and autophagy-modulating properties [36,37]. However, our knowledge regarding the effect that EOs or their constituents have on α-syn aggregation is rather limited, and the existing information is contradictory. While *Myrtus communis* (common Myrtle) EO increases the rate of α-syn fibrillation [38], the *Rosa Setate* x *Rosa Rugosa* (Kushui rose) EO [35], as well as cuminaldehyde, the main constituent of *Cuminum cyminum* (cumin) EO [39], has the opposite effect.

In this work, we constructed a whole-cell biosensor based on the split-luciferase complementation assay [40,41] and used it to investigate the anti-amyloidogenic effect of EOs from plants that are commonly used in the food industry in various settings. The split-luciferase complementation assay is a method to study protein–protein interactions via the reconstitution of a reporter luciferase enzyme [42]. The gene of a luciferase is appropriately fragmented into two parts, which in turn are fused to potentially interacting proteins. If these proteins interact, the luciferase fragments come to close proximity. As a result, the luciferase activity is restored and is detected through the catalysis of a light-emitting chemical reaction. Biosensors based on split-luciferase complementation assays have been used to test for protein–protein interactions in several settings, including ligand–receptor binding [43], receptor dimerization [44] and function [45], channel–protein interactions [46], kinase interactions [47] and signaling [48], apoptosis [49], viral infection [50] and protein oligomerization [51,52,53,54]. By employing a biosensor to detect α-syn aggregation, we screened eight EOs: *Citrus medica* (citron), *Salvia officinalis* (sage), *Mentha spicata* (spearmint), *Thymbra capitata* (conehead thyme), *Mentha piperita* (peppermint), *Origanum onites* (oregano), *Origanum vulgare* (wild marjoram) and *Origanum dictamnus* (dittany). Citron and sage EOs, as well as two of their major constituents, linalool and eucalyptol, showed significant reduction against α-syn aggregation, demonstrating that these natural compounds possess promising anti-aggregative properties and can be considered in complementary therapies for improving the quality of life of PD patients.

## 2. Materials and Methods

### 2.1. Essential Oils and Constituent Compounds

All the EOs were obtained from plants grown in Greece. The *Citrus medica* EO was supplied by VIORYL S.A. (Chemical and Agricultural Industry, Scientific Research S.A, Afidnes, Greece). The EOs of *Salvia officinalis*, *Mentha spicata*, *Thymbra capitata*, *Mentha piperita*, *Origanum onites*, *Origanum vulgare* and *Origanum dictamnus* were purchased from Provata (Athens, Greece). (*R*)-(+)-Limonene, (*S*)-(−)-limonene, linalool and eucalyptol were purchased from Sigma-Aldrich (Burlington,, MA, USA). Stock solutions of 10 mM of (*R*)-(+)-limonene, (*S*)-(−)-limonene, linalool and eucalyptol were prepared in ethanol and diluted prior to use in Dulbecco’s MEM (DMEM) (Biosera, Nuaille, France) in the desired concentration.

### 2.2. Plasmids

All expression constructs were made in the pGL3-CMV-Init plasmid, in which the transcription is driven by the human CMV promoter [55]. Using this vector, we prepared the following constructs:

(a) C1: It contains the sequence encoding the α-syn open reading frame (ORF) fused with a 30 bp linker, followed by the sequence encoding the aminoterminal part (amino acids 1–93) of *Gaussia princeps* luciferase (GLuc) protein, as described in [31].
(b) C2: It contains the sequence encoding the α-syn ORF fused with a 30 bp linker, followed by the sequence encoding the carboxyterminal part (amino acids 94–169) of GLuc protein, as described in [31].
(c) C3: It contains the sequence encoding the GLuc protein (amino acids 1–169).

Finally, pFLuc, a plasmid expressing the Firefly Luciferase under the control of the human EF1A promoter, was also generated and used in the transfection assays for normalization purposes.

The inserts for C1, C2 and C3 were generated by gene synthesis (Eurofins, Luxembourg). The sequences of all plasmids were verified by Sanger sequencing (Starseq, Mainz, Germany), and the exact sequence of their expressing cassettes is presented in Supplementary Figure S1.

### 2.3. Cell Culture, Transfection and Treatments

HeLa cells were routinely cultured in DMEM (Biosera, Nuaille, France) containing 10% *v*/*v* fetal bovine serum (Gibco, Waltham, MA, USA), 100 U/mL penicillin and 0.1 mg/mL streptomycin (Biosera, Nuaille, France) and 2 mM glutamine (Biosera, Nuaille, France) and maintained at 37 °C in a humidified incubator with 5% *v*/*v* CO_2_/ 95% *v*/*v* air. Cells were seeded in 96-well plates (Corning, New York, NY, USA) at a density of 8000 cells per well and the next day were transfected with 50 ng DNA using the JetPRIME reagent according to the manufacturer’s instructions (Polyplus, Illkirch-Graffenstaden, France). In all transfections, 5 ng of the pFLuc plasmid used for normalization was also added. Then, 24 h after transfection, the EOs or their constituents were diluted in DMEM at a specific dilution, and 10 μL was dispensed into each well. Cells were incubated for 48 h, and then the supernatant (the cell medium) was collected for GLuc assays, while the cells were lysed for FLuc assays. GLuc and FLuc assays were performed as described below.

### 2.4. Gaussia Luciferase Assay

GLuc bioluminescence was measured 72 h post transfection, employing the Pierce Gaussia Luciferase Glow Assay Kit (Thermo Scientific, Waltham, MA, USA) according to the manufacturer’s instructions. Briefly, 20 μL of supernatant from each well was transferred into a flat, white, opaque, 96-well plate (Thermo Scientific, Waltham, MA, USA) and mixed with 50 μL of fresh working solution (i.e., 49.5 μL Gaussia Glow Assay buffer and 0.5 μL 100× Coelenterazine) at room temperature. Bioluminescence (RLU) was measured immediately (integration time 2000 milliseconds) in an Infinite F200 Pro luminometer (Tecan, Männedorf, Switzerland).

### 2.5. Firefly Luciferase Assay

Firefly luciferase was used for the normalization of the GLuc bioluminescence measurements. For the FLuc assays, cells were rinsed with 150 μL 1×PBS and incubated in 150 μL of lysis buffer (25 mM Tris buffer pH 7.8, 2 mM EDTA, 1% Triton X-100, 10% Glycerol, 2 mM Dithiothreitol) under shaking for 30 min at room temperature. Then, 20 μL of lysate was transferred into a white, opaque, 96-well plate, and 80 μL of working solution [i.e., 50 μL Luciferase Assay Buffer, (40 mΜ Tricine pH7.8, 66.6 mM DTT, 1 mM ATP, 5.3 mM MgSO_4_, 0.2 mM EDTA, 540 mM coenzyme A), 25 μL ddH_2_O and 5 μL 20× D-Luciferin Potassium Salt (Regis Technologies, Morton Grove, IL, USA)] was added at room temperature. Bioluminescence (RLU) was measured (integration time 2000 milliseconds) in an Infinite F200 Pro luminometer (Tecan, Männedorf, Switzerland).

### 2.6. Normalization and Statistical Analysis

Normalization was performed by dividing the RLU value of GLuc of each well by the RLU value of FLuc of the same well. Each experiment included at least two technical replicates and was repeated at least thrice. Results were expressed as mean ± SD. Statistical analyses were performed by one-way ANOVA for multiple comparisons and by student’s test for the comparison between two groups, using GraphPad Prism 10.1.2. Differences were considered significant at *p* ≤ 0.01.

## 3. Results

### 3.1. Construction and Validation of a Split-Gaussia Luciferase Complementation Assay to Assess α-Syn Aggregation

The prior successful use of GLuc-based biosensors in the study of protein oligomerization, both in live cells [51,52,54] and animals [53], prompted us to construct a biosensor in an effort to study the effect of EOs on α-syn aggregation in live cells. As a luciferase enzyme, we selected *Gaussia princeps* luciferase, which, compared to other luciferases, is characterized by high sensitivity, thus allowing for the detection of low levels of luciferase activity; this is particularly advantageous, as in the complementation assays, only a percentage of the activity of the intact enzyme is restored. Additionally, GLuc is a stable enzyme with a broad dynamic range, allowing for the quantitation of both low and high levels of activity; therefore, it is suitable for various experimental conditions and applications.

**Figure 1:**
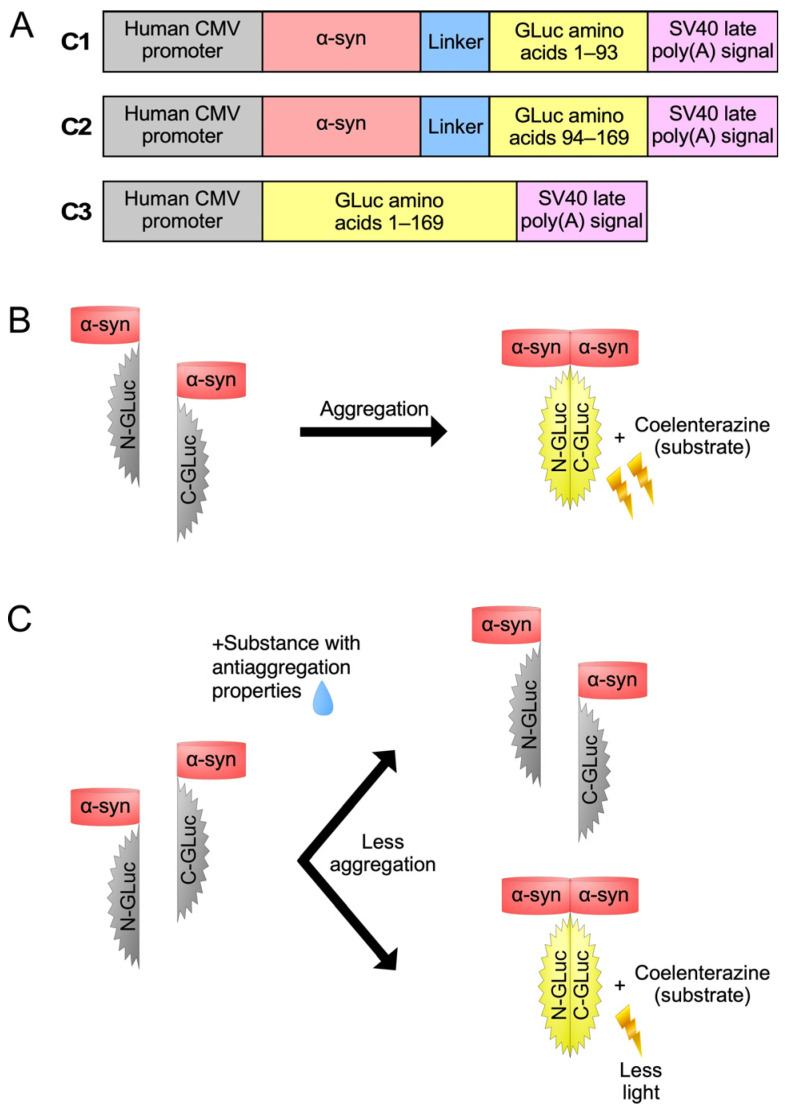
The split-*Gaussia* luciferase whole-cell biosensor utilized in the present study. (**A**) Schematic representation of the expression cassettes of the three plasmid constructs (C1, C2 and C3) used in this study. (**B**) Co-transfection with constructs C1 and C2 results in GLuc activity. (**C**) Following C1/C2 co-transfection, the GLuc activity is reduced upon treatment with substances that have anti-aggregating properties.

The principle of function of our whole-cell biosensor is depicted in Figure 1. The C1 plasmid construct expresses the human α-syn fused with the aminoterminal part of GLuc protein, while the C2 expresses the human α-syn fused with the carboxyterminal part of GLuc protein (Figure 1A). The C3 expresses the non-split, functional GLuc protein and was used as a positive control. When α-syn monomers interact with each other, GLuc enzymatic activity is restored and can be detected by light emission in the presence of its substrate, coelenterazine (Figure 1B). However, when a substance with anti-aggregating properties is added (Figure 1C), a reduction in the restored GLuc activity is observed.

**Figure 2:**
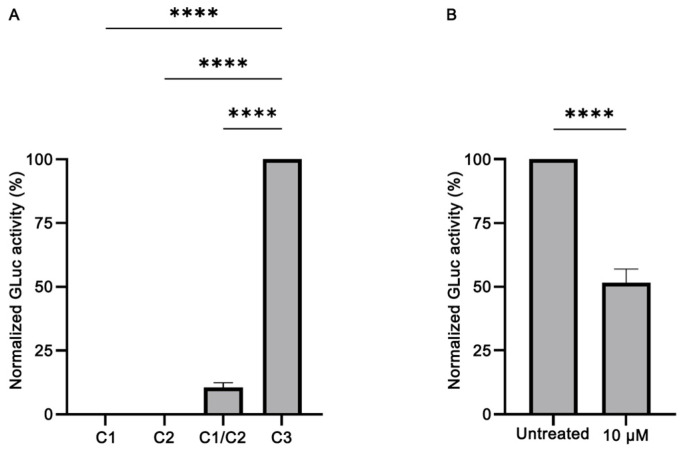
Validation of the split-Gaussia luciferase whole-cell biosensor in HeLa cells. (**A**) Transfection with C1 or C2 plasmid constructs does not elicit GLuc activity. However, upon co-transfection with C1 and C2 (at 1:1 molecular ratio), the activity of GLuc was partly restored, reaching ~10% of the activity of the non-split, functional GLuc protein (C3) (*n* = 4, **** *p* < 0.0001). The GLuc activity of the positive control C3 construct was set as 100%. (**B**) Treatment with baicalein (at 10 μM final concentration), a known inhibitor of α-syn aggregation, led to a reduction of the GLuc activity obtained by C1/C2 co-transfection by ~50% (*n* = 7, **** *p* < 0.0001). The GLuc activity of the untreated sample was set as 100%.

To evaluate our biosensor, we transfected HeLa cells with C1 and C2 alone or in combination and tested for GLuc activity 72 h post transfection. Transfection with the GLuc expressing C3 plasmid was used as positive control. In addition, in the transfection experiments, the pFLuc plasmid was co-transfected, and the Firefly luciferase activity was used for normalization purposes. Our results (Figure 2A) showed that our biosensor works efficiently: although transfection with C1 or C2 alone does not elicit GLuc activity (*p* < 0.0001), upon co-transfection with C1 and C2 (at 1:1 molecular ratio), the activity of GLuc was partly restored, reaching ~10% of the activity of the non-split, functional GLuc protein (*p* < 0.0001). This finding reveals that the levels of bioluminescence emitted by the restored GLuc activity were sufficiently high to proceed with the evaluation of the potential anti-amyloidogenic properties of the EOs. Moreover, the addition of baicalein, a known inhibitor of α-syn aggregation, led to a reduction of the GLuc activity obtained by C1/C2 co-transfection by ~50% (*p* < 0.0001) (Figure 2B), as previously shown [56,57]. Given that α-syn can be excreted from the cells [57,58,59,60,61], following this initial characterization, we also tested for GLuc activity in the supernatants of the cells. Notably, high levels of GLuc activity were detected in the supernatant of the cultures, approximately 10× higher that in the lysates (Supplementary Figure S2). Thus, in the following experiments, GLuc activity was measured only in the supernatant.

### 3.2. Assessing the Effect of EOs on α-Syn Aggregation

**Figure 3:**
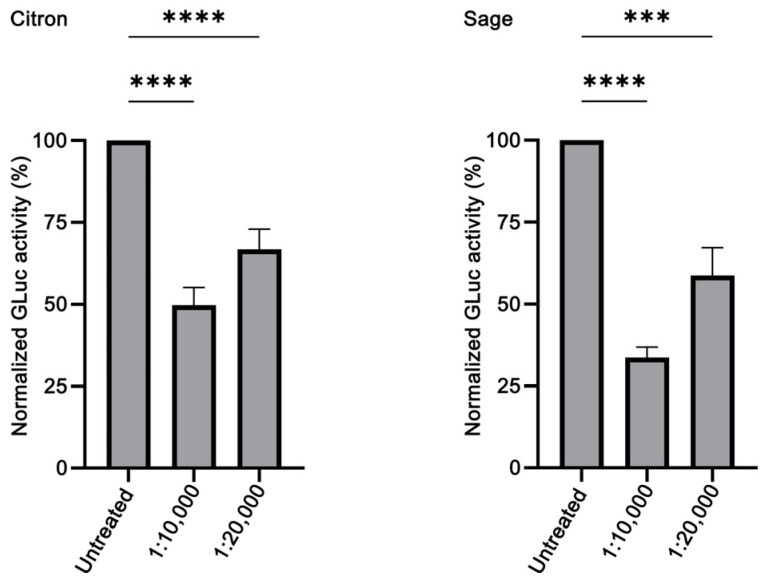
Evaluation of the anti-aggregating properties of *Citrus medica* (citron) and *Salvia officinalis* (sage) EOs in HeLa cells. The restored GLuc activity obtained by C1/C2 co-transfection is reduced in a concentration-dependent manner upon treatment with both citron (*n* = 4, **** *p* < 0.0001) and sage (*n* = 3, *** *p* ≤ 0.001, **** *p* < 0.0001) EOs at the indicated dilutions. The GLuc activity of the untreated sample was set as 100%.

Given that numerous studies have shown that EOs and their components have neuroprotective effects (reviewed in [62]), we decided to evaluate the effect against α-syn aggregation of commonly used EOs using our biosensor. We screened in HeLa cells the EOs of *C. medica* (citron), *S. officinalis* (sage), *M. spicata* (spearmint) and *T. capitata* (conehead thyme), for which the major constituents are known [63,64,65,66], and the EOs of *M. piperita* (peppermint), *O. onites* (oregano), *O. vulgare* (wild marjoram) and *O. dictamnus* (dittany) at dilutions ranging from 1:10,000 to 1:50,000. Two of them, *C. medica* and *S. officinalis*, showed anti-amyloidogenic properties. The GLuc activity following C1/C2 co-transfection was reduced upon treatment with C. medica EO; 1:10,000 dilution resulted in ~50% reduction (*p* < 0.0001), while 1:20,000 (*p* < 0.0001) in ~30% reduction, indicating that this EO can inhibit α-syn aggregation in a concentration-dependent fashion (Figure 3). Similar results were obtained with *S. officinalis* EO, as shown in Figure 3. Following C1/C2 co-transfection, cells were treated with two different dilutions of *S. officinalis* EO, namely, 1:10,000 and 1:20,000, which reduced the GLuc activity by ~60% (*p* < 0.0001) and ~40% (*p* < 0.001), respectively. It should be noted that the citron and sage EOs dilutions employed were the lowest ones at which there was an effect on α-syn aggregation.

### 3.3. Assessing the Effect of Major Constituents of Citron and Sage EOs on α-Syn Aggregation

Next, we sought to identify the constituent components thanks to which the assessed EOs exhibit anti-aggregating properties. We hypothesized that the anti-amyloidogenic constituents must be among the compounds that are present in higher concentrations in the EOs we tested. Such compounds are limonene and linalool, comprising approximately 38% *v*/*v* and 35% *v*/*v* of citron EO, respectively [63], while eucalyptol (1,8-cineole) is the major ingredient in sage EO, with a percentage of approximately 43% *v*/*v* [64]. The structure of these compounds is presented in Table 1.

**Figure 4:**
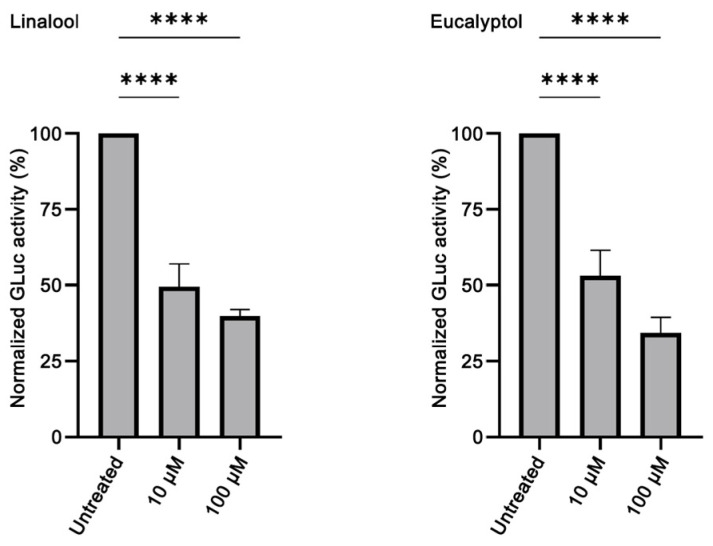
Evaluation of the anti-aggregating properties of linalool and eucalyptol in HeLa cells. The restored GLuc activity obtained by C1/C2 co-transfection is reduced in a concentration-dependent manner upon treatment with both linalool (*n* = 4, **** *p* < 0.0001) and eucalyptol (*n* = 3, **** *p* < 0.0001) at the indicated dilutions. The GLuc activity of the untreated sample was set as 100%.

The GLuc activity following C1/C2 co-transfection of HeLa cells was reduced upon treatment with linalool diluted in DMEM from a 10 mM stock in absolute ethanol. At 10 μM, the observed reduction was ~50% (*p* < 0.0001), while at 100 μM, it was ~65% (*p* < 0.0001) (Figure 4). Limonene, however, did not affect α-syn aggregation. It should be noted that limonene can occur as two enantiomeric forms, that is, R(+)-limonene and S(−)-limonene, with the former one being the enantiomer usually found in plants [67]. Although both limonene enantiomers were tested for their anti-amyloidogenic effect, none of them was shown to have the ability to inhibit α-syn aggregation (Supplementary Figure S3).

Anti-aggregation activity was also observed with eucalyptol diluted in DMEM from a 10 mM stock in absolute ethanol. The GLuc activity following C1/C2 co-transfection was reduced upon treatment with 10 μM or 100 μM eucalyptol by ~50% (*p* < 0.0001) and ~75% (*p* < 0.0001), respectively (Figure 4).

## 4. Discussion

α-Syn misfolding and aggregation are considered to play a key role in the pathogenesis of synucleinopathies, a series of neurodegenerative diseases including PD. PD and related disorders are characterized histopathologically by intraneuronal inclusions known as Lewy bodies and Lewy neurites [68,69], which are predominantly composed of fibrillar α-syn [70,71]. Additionally, most of the identified α-syn disease-inducing mutations (A30P, A53T/E/V, E46K, H50Q, G51D) have been shown to promote the aggregation of the protein (reviewed by [72]), while in vivo studies indicate that α-syn aggregation promotes neurotoxicity and neurodegeneration [73,74]. However, the findings regarding the toxic α-syn species have been inconclusive [75], and on the top of that, it remains unclear how α-syn aggregation promotes toxicity. Yet, inhibition of α-syn aggregation constitutes an attractive therapeutic strategy. Thus, searching for aggregation inhibitors among natural compounds appears increasingly promising, especially in light of the fact that a natural compound [epigallocatechin gallate (EGCG)] has entered clinical trials as a potential anti-aggregating treatment against a synucleinopathy, Multiple System Atrophy (MSA) [76].

Biosensors based on the split-luciferase complementation assay have been used in the study of protein–protein interactions in several settings, ranging from receptor structure and function to cell apoptosis and protein oligomerization. Indeed, this approach has recently emerged as a useful tool for assessing various neurodegeneration-related aggregating proteins, such as α-syn [40,51,77,78], Aβ [46], tau protein [79,80] and the transactivating response region DNA Binding Protein (TDP-43) [81] in live cells, in vivo [53,82,83,84] and ex vivo [85].

In this context, we constructed a biosensor, which we used to investigate the effect of several EOs on α-syn aggregation in live cells. *Gaussia princeps* luciferase was the enzyme of choice, as it presents certain advantages compared to luciferases of other organisms: it is a monomeric protein of 19.9 kDa, the smallest coelenterazine-using luciferase that does not require ATP to be active, and its humanized form can generate an over 100× higher bioluminescent signal compared to the humanized forms of Firefly and Renilla luciferases [41]. This is particularly advantageous, as in the complementation assays, only a percentage of the activity of the intact enzyme is restored. To test and validate our biosensor, we compared the restored GLuc activity to that of the non-split, fully functional GLuc protein. As shown in Figure 2A, the α-syn monomers interaction restored ~10% of the GLuc activity. Additionally, when baicalein, a known potent inhibitor of α-syn aggregation [56], was added following transfection, a reduction in the restored GLuc activity by ~50% was observed (Figure 2B), consistent with previous works [56,57].

Out of the eight EOs that we tested, two exhibited anti-amyloidogenic properties. Citron EO, at 1:10,000 and 1:20,000 dilutions, reduced restored GLuc activity by ~50% and ~30%, respectively (Figure 3). To the best of our knowledge, this is the first time that *C. medica* EO has been assessed for its anti-amyloidogenic effect, as there is no previous retrievable published work evaluating citron EO on amyloid aggregation. Since ancient times, citron, which was possibly the only citrus fruit known in Europe, was known among others for its digestive, antitoxic and insect-repelling properties [86]. Modern literature has confirmed some of these properties and has revealed new ones, such as antimicrobial, antidiabetic, anti-inflammatory, antitumor and cardioprotective aspects [87]. Given that these citron properties are largely due to its various bioactive compounds (flavonoids, terpenes, carotenoids), we seek to assess the major compounds (limonene and linalool [63]) in our citron EO for their anti-aggregating properties. Although it could be expected for limonene, as a phenolic compound, to be able to prevent aggregation [88], in our hands, limonene did not show any effect at any concentration used. This finding is in accordance with a previous study showing that, despite the neuroprotective effect of limonene on an AD *Drosophila* model, it did not have any effect on Aβ_42_ aggregation [89]. Linalool, on the other hand, proved to be a good aggregation inhibitor, inducing at the highest concentration used more than 50% reduction of the restored GLuc activity (Figure 4). Although linalool has never been tested before against α-syn aggregation, it has been shown to decrease Aβ_40_ and Aβ_42_ levels in the brain of 3×Tg-AD mice [11].

Sage EO also displayed good inhibiting potential at 1:10,000 and 1:20,000 dilutions; it reduced restored GLuc activity by ~40% and ~60%, respectively. Sage, a culinary herb and a well-known medicinal plant, has become recently known as a cognition-enhancing, neuroprotective and anti-AD agent (reviewed in [90,91,92]). Sage EO has never been reported for its anti-amyloidogenic properties, although the *S. officinalis* extract has been lately shown to decrease the deposition of Aβ in scopolamine-treated rats [93]. Ιn this study, eucalyptol, the main component in sage EO, similarly to sage EO, induced a decrease in the restored GLuc activity (Figure 4). As far as we can tell, there are no prior reports on eucalyptol modulating α-syn aggregation, but it has been demonstrated that eucalyptol can inhibit the in vitro oligomerization of Aβ_42_ [94].

Translating an in vitro dose determined in a cell culture system to an appropriate dose for pre-clinical or clinical studies is a very challenging task. However, it is worth mentioning that oral administration of 25 mg/kg of linalool every 48 h for 3 months had a series of positive effects in an AD transgenic mouse model [11]. Similarly, in a rat AD model, administration of 100–150 mg/kg of eucalyptol daily for 28 days had also positive effects [95]. Thus, in the AD context, both linalool and eucalyptol can have a therapeutic value at doses tolerated by the animals.

## 5. Conclusions

The present study provides new insights into the properties of two EOs used in food and the hygienic industry. Both citron and sage EOs, as well as their main constituents, linalool and eucalyptol, inhibited α-syn aggregation in a concentration-dependent manner, exhibiting promising anti-amyloidogenic properties. Notably, the reduction of restored GLuc activity observed with linalool is comparable to that of citron EO. Moreover, the reduction of restored GLuc activity observed with eucalyptol is comparable to that achieved by sage EO. These results suggest that in the citron and sage EOs, linalool and eucalyptol are, most likely, the sole constituents affecting α-syn aggregation. However, more experiments are needed to further validate their effectiveness, while in silico molecular docking studies can also contribute towards this direction.

Apart from their ability to cross the blood–brain barrier [96,97], the fact that linalool and eucalyptol can be taken orally has an added value, as there is now increasing evidence that α-syn pathology appears in the gastrointestinal tract and enteric nervous system well before it makes its entrance in the brain [98]. In vivo studies of citron and sage essential oils, along with their primary components, linalool and eucalyptol, are the next logical step for further assessment of their anti-amyloidogenic properties. Furthermore, it would be very interesting to explore their use as ingredients in functional foods or in complementary therapies for PD.

## Figures and Tables

**Table 1:** Structure and percentage presence of tested compounds in the *C. medica* and *S. officinalis* EOs, as estimated by GC/MS analysis in Mitropoulou et al., 2020 and Vetas et al., 2017 [63,64].

Name	Structure	% in Citron EO	% in Sage EO
Linalool	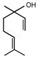	35.44	0.45
(R)-(+)-Limonene	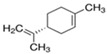	38.46	-
S)-(−)-Limonene	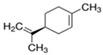	-	-
Eucalyptol	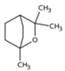	-	43.06

## Data Availability

The authors confirm that the data supporting the findings of this study are available within the article and/or its Supplementary Materials.

## Supplementary Materials

The following supporting information can be downloaded at: https://www.mdpi.com/article/10.3390/brainsci14010035/s1. Supplementary Figure S1. The expression cassettes of the four plasmid constructs used in this study. In the C1, C2 and C3 constructs the expression is driven by the human CMV promoter. In the pFLuc construct the expression is driven by the human EF1A promoter. The sequences encoding α-syn are indicated in bold. The sequences encoding the luciferases (GLuc or FLuc) are indicated in uppercase. The start and the stop codons are underlined. In the constructs C1 and C2 the sequence which encodes the flexible linker that separates a-syn from the GLuc part is indicated in italics. Supplementary Figure S2. GLuc activity in the supernatant (SN) and lysate (LYS) of HeLa cells co-transfected with C1/C2. Approximately 10× higher levels of GLuc activity were detected in the supernatant. (*n* = 3, **** *p* < 0.001). Supplementary Figure S3. Evaluation of the anti-aggregating properties of R(+)-limonene or S(−)-limonene in HeLa cells. The restored GLuc activity obtained by C1/C2 co-transfection was not affected even at 100 μM limonene (diluted in DMEM from an initial stock in absolute ethanol) (*n* = 4, ns = non significance difference). The GLuc activity of the untreated sample was set as 100%.
