# Supplementary material for: Assessment of the Anti-Amyloidogenic Properties of Essential Oils and Their Constituents in Cells Using a Whole-Cell Recombinant Biosensor
Source: Brain Sci. 2023 Dec 29;14(1):35. doi: 10.3390/brainsci14010035 (PMC10812981; doi:10.3390/brainsci14010035)
Supplement: Supplementary file 1 [file brainsci-14-00035-s001.zip › brainsci-2778059-supplementary.pdf]

C1

5′_[CMV_promoter]**atggatgtattcatgaaaggactttcaaaggccaaggagggagttgtggctgctgctgagaaaaccaaac**
agggtgtggcagaagcagcaggaaagacaaaagagggtgttctctatgtaggctccaaaaccaaggagggagtggtgcatggtgtg
gcaacagtggctgagaagaccaaagagcaagtgacaaatgttggaggagcagtggtgacgggtgtgacagcagtagcccagaagac
agtggagggagcagggagcattgcagcagccactggctttgtcaaaaaggaccagttgggcaagaatgaagaaggagccccacagg
aaggaattctggaagatatgcctgtggatcctgacaatgaggcttatgaaatgccttctgaggaagggtatcaagactacgaacct
gaagcc*ggcggagggggaagcggaggagggggctcc*ATGAAGCCCACCGAGAACAACGAAGACTTCAACATCGTGGCCGTGGCCAG
CAACTTCGCGACCACGGATCTCGATGCTGACCGCGGGAAGTTGCCCGGCAAGAAGCTGCCGCTGGAGGTGCTCAAAGAGATGGAAG
CCAATGCCCGGAAAGCTGGCTGCACCAGGGGCTGTCTGATCTGCCTGTCCCACATCAAGTGCACGCCCAAGATGAAGAAGTTCATC
CCAGGACGCTGCCACACCTACGAAGGCGACAAAGAGTCCGCACAGGGCGGCATAGGCtaa[SV40 polyA signal]_3′

C2

5′_[CMV_promoter]**atggatgtattcatgaaaggactttcaaaggccaaggagggagttgtggctgctgctgagaaaaccaaac**
agggtgtggcagaagcagcaggaaagacaaaagagggtgttctctatgtaggctccaaaaccaaggagggagtggtgcatggtgtg
gcaacagtggctgagaagaccaaagagcaagtgacaaatgttggaggagcagtggtgacgggtgtgacagcagtagcccagaagac
agtggagggagcagggagcattgcagcagccactggctttgtcaaaaaggaccagttgggcaagaatgaagaaggagccccacagg
aaggaattctggaagatatgcctgtggatcctgacaatgaggcttatgaaatgccttctgaggaagggtatcaagactacgaacct
gaagcc*ggcggagggggaagcggaggagggggctcc*GAGGCGATCGTCGACATTCCTGAGATTCCTGGGTTCAAGGACTTGGAGCC
CATGGAGCAGTTCATCGCACAGGTCGATCTGTGTGTGGACTGCACAACTGGCTGCCTCAAAGGGCTTGCCAACGTGCAGTGTTCTG
ACCTGCTCAAGAAGTGGCTGCCGCAACGCTGTGCGACCTTTGCCAGCAAGATCCAGGGCCAGGTGGACAAGATCAAGGGGGCCGGT
GGTGACtaa[SV40 polyA signal]_3′

C3

5′_[CMV_promoter]ATGAAGCCCACCGAGAACAACGAAGACTTCAACATCGTGGCCGTGGCCAGCAACTTCGCGACCACGGATC
TCGATGCTGACCGCGGGAAGTTGCCCGGCAAGAAGCTGCCGCTGGAGGTGCTCAAAGAGATGGAAGCCAATGCCCGGAAAGCTGGC
TGCACCAGGGGCTGTCTGATCTGCCTGTCCCACATCAAGTGCACGCCCAAGATGAAGAAGTTCATCCCAGGACGCTGCCACACCTA
CGAAGGCGACAAAGAGTCCGCACAGGGCGGCATAGGCGAGGCGATCGTCGACATTCCTGAGATTCCTGGGTTCAAGGACTTGGAGC
CCATGGAGCAGTTCATCGCACAGGTCGATCTGTGTGTGGACTGCACAACTGGCTGCCTCAAAGGGCTTGCCAACGTGCAGTGTTCT
GACCTGCTCAAGAAGTGGCTGCCGCAACGCTGTGCGACCTTTGCCAGCAAGATCCAGGGCCAGGTGGACAAGATCAAGGGGGCCGG
TGGTGACtaa[SV40 polyA signal]_3′

pFLuc

5′_[EF1A_promoter]ATGGAAGACGCCAAAAACATAAAGAAAGGCCCGGCGCCATTCTATCCGCTGGAAGATGGAACCGCTGGA
GAGCAACTGCATAAGGCTATGAAGAGATACGCCCTGGTTCCTGGAACAATTGCTTTTACAGATGCACATATCGAGGTGGACATCAC
TTACGCTGAGTACTTCGAAATGTCCGTTCGGTTGGCAGAAGCTATGAAACGATATGGGCTGAATACAAATCACAGAATCGTCGTAT
GCAGTGAAAACTCTCTTCAATTCTTTATGCCGGTGTTGGGCGCGTTATTTATCGGAGTTGCAGTTGCGCCCGCGAACGACATTTAT
AATGAACGTGAATTGCTCAACAGTATGGGCATTTCGCAGCCTACCGTGGTGTTCGTTTCCAAAAAGGGGTTGCAAAAAATTTTGAA
CGTGCAAAAAAAGCTCCCAATCATCCAAAAAATTATTATCATGGATTCTAAAACGGATTACCAGGGATTTCAGTCGATGTACACGT
TCGTCACATCTCATCTACCTCCCGGTTTTAATGAATACGATTTTGTGCCAGAGTCCTTCGATAGGGACAAGACAATTGCACTGATC
ATGAACTCCTCTGGATCTACTGGTCTGCCTAAAGGTGTCGCTCTGCCTCATAGAACTGCCTGCGTGAGATTCTCGCATGCCAGAGA
TCCTATTTTTGGCAATCAAATCATTCCGGATACTGCGATTTTAAGTGTTGTTCCATTCCATCACGGTTTTGGAATGTTTACTACAC
TCGGATATTTGATATGTGGATTTCGAGTCGTCTTAATGTATAGATTTGAAGAAGAGCTGTTTCTGAGGAGCCTTCAGGATTACAAG
ATTCAAAGTGCGCTGCTGGTGCCAACCCTATTCTCCTTCTTCGCCAAAAGCACTCTGATTGACAAATACGATTTATCTAATTTACA
CGAAATTGCTTCTGGTGGCGCTCCCCTCTCTAAGGAAGTCGGGGAAGCGGTTGCCAAGAGGTTCCATCTGCCAGGTATCAGGCAAG
GATATGGGCTCACTGAGACTACATCAGCTATTCTGATTACACCCGAGGGGGATGATAAACCGGGCGCGGTCGGTAAAGTTGTTCCA
TTTTTTGAAGCGAAGGTTGTGGATCTGGATACCGGGAAAACGCTGGGCGTTAATCAAAGAGGCGAACTGTGTGTGAGAGGTCCTAT
GATTATGTCCGGTTATGTAAACAATCCGGAAGCGACCAACGCCTTGATTGACAAGGATGGATGGCTACATTCTGGAGACATAGCTT
ACTGGGACGAAGACGAACACTTCTTCATCGTTGACCGCCTGAAGTCTCTGATTAAGTACAAAGGCTATCAGGTGGCTCCCGCTGAA
TTGGAATCCATCTTGCTCCAACACCCCAACATCTTCGACGCAGGTGTCGCAGGTCTTCCCGACGATGACGCCGGTGAACTTCCCGC
CGCCGTTGTTGTTTTGGAGCACGGAAAGACGATGACGGAAAAAGAGATCGTGGATTACGTCGCCAGTCAAGTAACAACCGCGAAAA
AGTTGCGCGGAGGAGTTGTGTTTGTGGACGAAGTACCGAAAGGTCTTACCGGAAAACTCGACGCAAGAAAAATCAGAGAGATCCTC
ATAAAGGCCAAGAAGGGCGGAAAGATCGCCGTGtaa[SV40 polyA signal]_3′

Supplementary Figure S1. The expression cassettes of the four plasmid constructs used in this study. In the C1, C2 and C3 constructs the expression is driven by the human CMV promoter. In the pFLuc construct the expression is driven by the human EF1A promoter. The sequences encoding α-syn are indicated in bold. The sequences encoding the luciferases (GLuc or FLuc) are indicated in uppercase. The start and the stop codons are underlined. In the constructs C1 and C2 the sequence which encodes the flexible linker that separates a-syn from the GLuc part is indicated in italics.

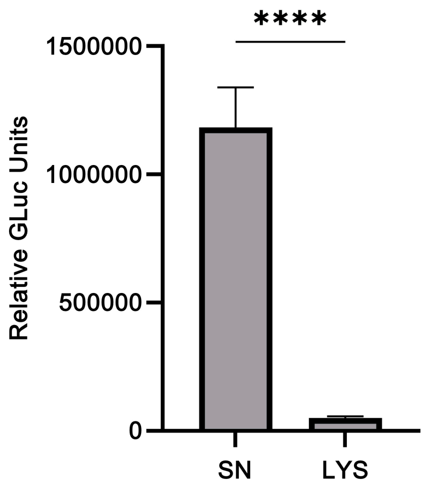

Supplementary Figure S2. GLuc activity in the supernatant (SN) and lysate (LYS) of HeLa cells co-transfected with C1/C2. Approximately 10x higher levels of GLuc activity were detected in the supernatant. (n=3, **** $p<0.001$).

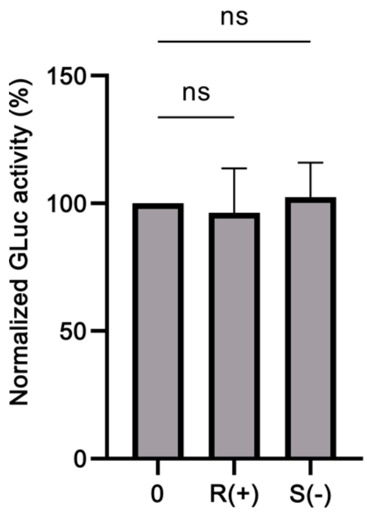

Supplementary Figure S3. Evaluation of the anti-aggregating properties of R(+)-limonene or S(-)-limonene in HeLa cells. The restored GLuc activity obtained by C1/C2 co-transfection was not affected even at 100 µM limonene (diluted in DMEM from an initial stock in absolute ethanol) (n= 4, ns = non significance difference). The GLuc activity of the untreated sample was set as 100 %.
